# Supplementary figures and images for: Combination of EphA2- and Wee1-Targeted Therapies in Endometrial Cancer
Source: Int J Mol Sci. 2023 Feb 15;24(4):3915. doi: 10.3390/ijms24043915 (PMC9962847; doi:10.3390/ijms24043915)

**A** **Figure S1**

Hec1A

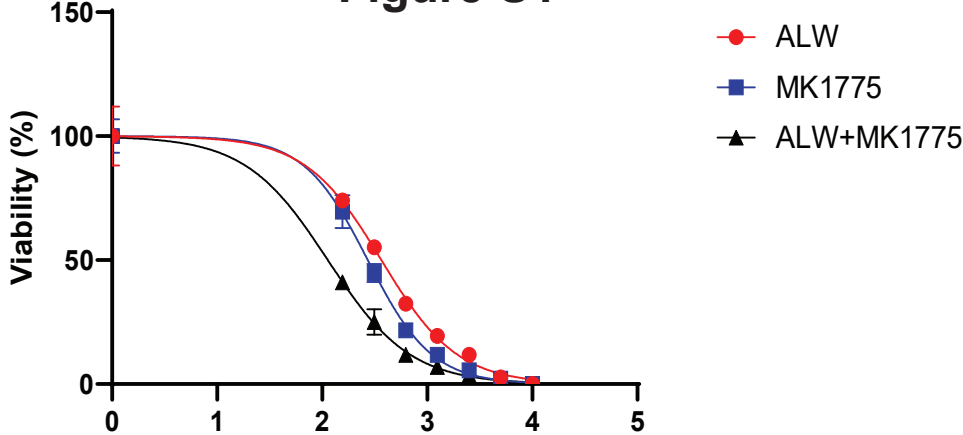

**B**

Ishikawa

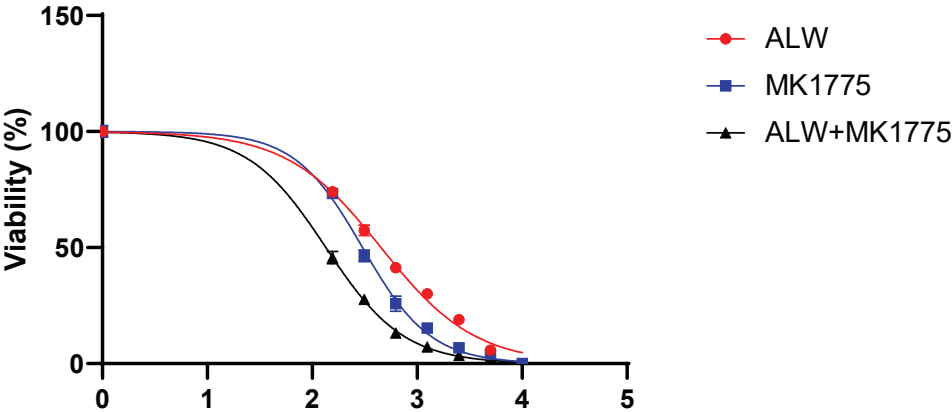

**C**

Hec1A 24H CELL CYCLE

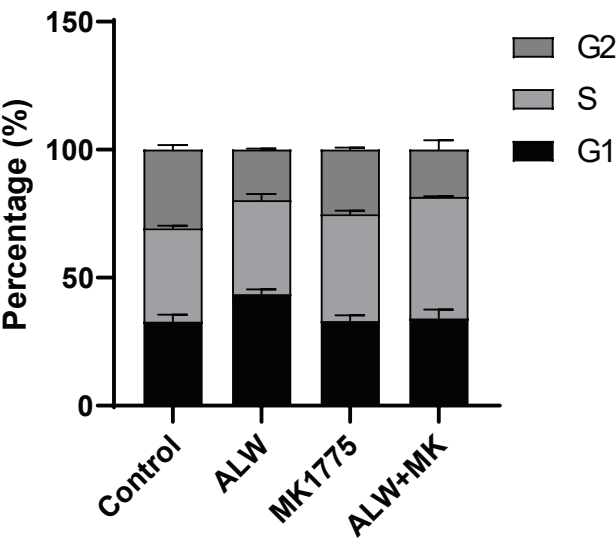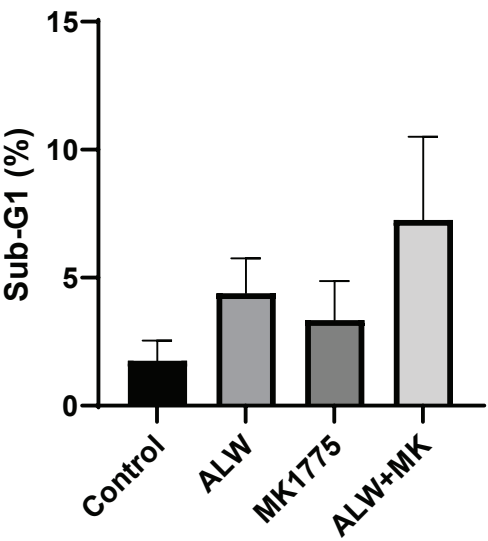

**D**

ISHIKAWA 24H CELL CYCLE

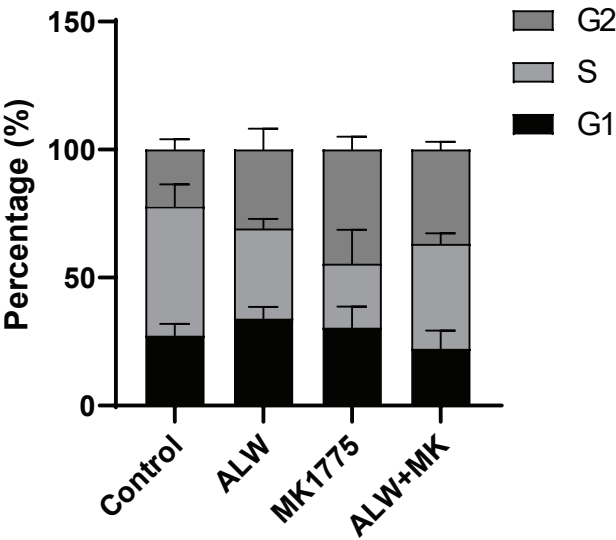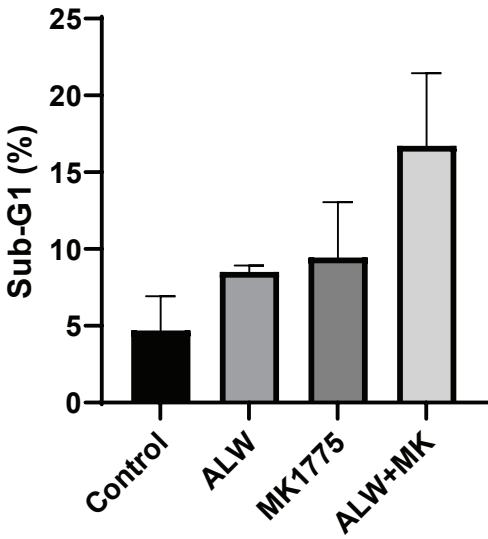

**Figure S2**

**A**

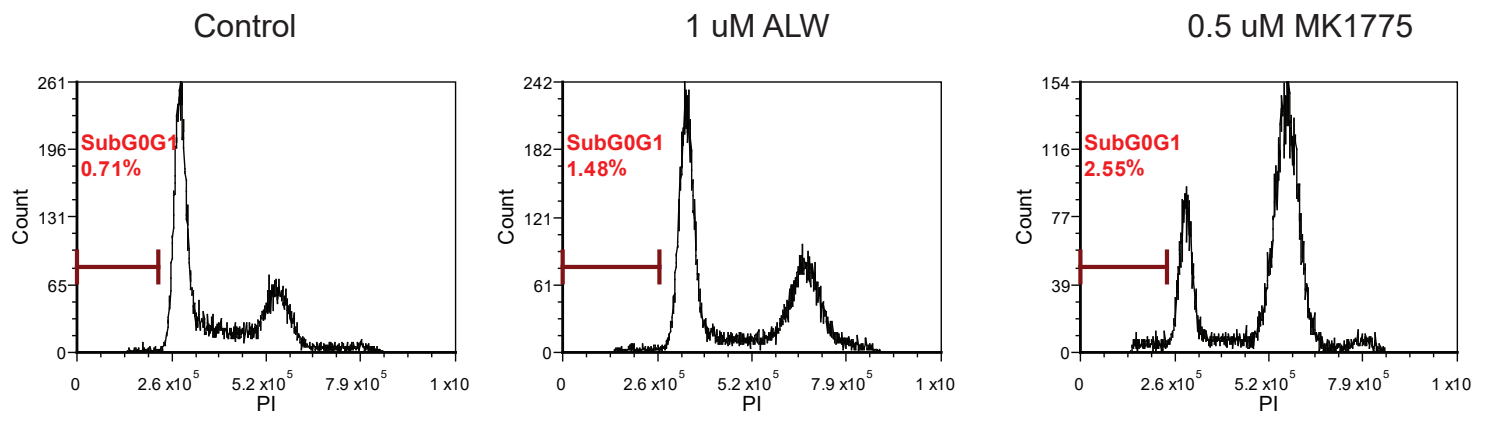

**B**

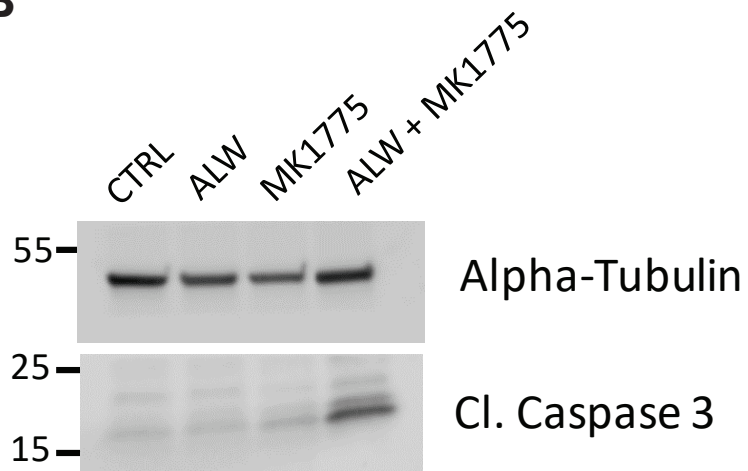

**C**

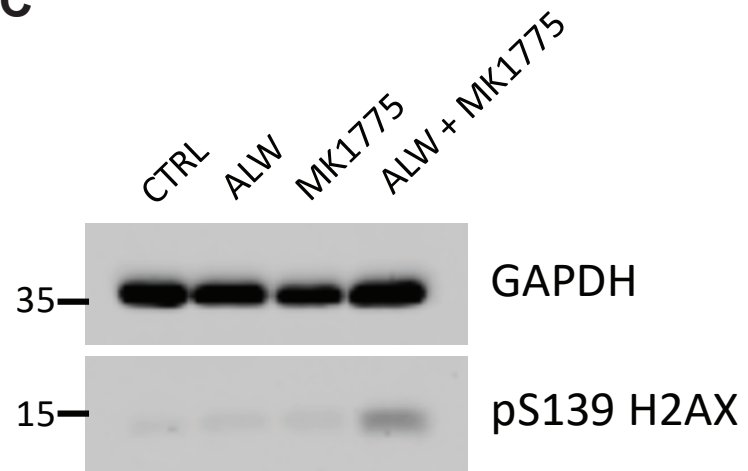

Supplement: Supplementary file 1 [file ijms-24-03915-s001.zip › ijms-2114008-supplementary.pdf]
